# Sustained Effectiveness of a Fixed-Dose Combination of Artesunate and Amodiaquine in 480 Patients with Uncomplicated* Plasmodium falciparum* Malaria in Côte d'Ivoire

**DOI:** 10.1155/2017/3958765

**Published:** 2017-12-07

**Authors:** Serge Brice Assi, Abouo Franklin Nguessan, Yapo Thomas Aba, André Offianan Toure, Hervé Menan, Jean Claude Yavo, Koffi Moïse San, Emmanuel Bissagnéné, Stephan Duparc, Valérie Lameyre, Mea Antoine Tanoh

**Affiliations:** ^1^Institut Pierre Richet (IPR)/Institut National de Santé Publique (INSP), Bouaké, Côte d'Ivoire; ^2^National Malaria Control Programme, Abidjan, Côte d'Ivoire; ^3^Infectious and Tropical Diseases Unit, Treichville University Hospital, Abidjan, Côte d'Ivoire; ^4^Infectious and Tropical Diseases Department, Bouaké University Hospital, Bouaké, Côte d'Ivoire; ^5^Institut Pasteur de Côte d'Ivoire, Unité de Paludologie, Abidjan, Côte d'Ivoire; ^6^Diagnostic and Research Center on AIDS and Other Infectious Diseases (CeDReS), Abidjan, Côte d'Ivoire; ^7^Pharmacovigilance Unit, Medical Sciences, Felix Houphouët-Boigny, Abidjan, Côte d'Ivoire; ^8^Medicines for Malaria Venture, Geneva, Switzerland; ^9^Sanofi Access to Medicines, Gentilly, France

## Abstract

The objective of this study was to monitor the effectiveness of artesunate-amodiaquine fixed-dose combination tablets (ASAQ Winthrop®) in the treatment of uncomplicated* Plasmodium falciparum* malaria in Côte d'Ivoire. Two enrolment periods (November 2009 to May 2010 and March to October 2013) were compared using an identical design. Subjects with proven monospecific* P. falciparum* infection according to the WHO diagnostic criteria were eligible. 290 patients during each period received a dose of ASAQ Winthrop tablets appropriate for their age. The primary outcome measure was PCR-corrected adequate clinical and parasitological response at Day 28 in the per protocol population (255 in Period 1 and 240 in Period 2). This was achieved by 95.7% of patients during Period 1 and 96.3% during Period 2. Over 95% of patients were afebrile at Day 3 and complete parasite clearance was achieved at Day 3 in >99% of patients. Nineteen adverse events in nineteen patients were considered as possibly related to treatment, principally vomiting, abnormal liver function tests, and pruritus. There was no evidence for loss of effectiveness over the three-year period in spite of strong drug pressure. This trial was registered in the US Clinical Trials Registry (clinical.trials.gov) under the identifier number NCT01023399.

## 1. Introduction

Artemisinin-based combination therapy (ACT) has become the standard for antimalarial therapy worldwide and is recommended by the World Health Organization (WHO) as first-line treatment for uncomplicated* Plasmodium falciparum* malaria since the beginning of the century [1–3]. In Côte d'Ivoire, the National Malaria Control Programme (NMCP) decided in 2007 to guarantee provision and delivery of ACT in all malaria treatment centres throughout the country. Artesunate-amodiaquine combinations were proposed as first-line treatment, and artemether-lumefantrine combinations as second-line treatment.

Despite widespread use of ACT since 2001, emergence of ACT resistance in Africa has not been convincingly demonstrated [4, 5]. The mutations associated with cases of artemisinin resistance in South-East Asia [6, 7] have not been documented in Africa to date. Although there is some evidence for reduced sensitivity to some of the partner drugs used in ACT, this has only been clearly demonstrated when these have been used in monotherapy [8, 9]. Nevertheless, it is important to remain vigilant for any signal of emerging resistance to ACT in sub-Saharan Africa. In this context, it is considered best practice to perform efficacy studies at representative sentinel sites every two years [10].

Between 2004 and 2007, a fixed-dose combined formulation containing artesunate (AS) and amodiaquine (AQ) in a single tablet (ASAQ Winthrop) was developed by Sanofi in partnership with the Drugs for Neglected Disease initiative (DNDi) [11]. This treatment has been demonstrated to be effective in clearing* P. falciparum* parasites from infected individuals in a large number of clinical trials [12–19]. At the time of the introduction of ASAQ Winthrop, Sanofi and the DNDi wanted to ensure that appropriate postmarketing data was available as quickly as possible on the safety and effectiveness of this antimalarial treatment in the field. To this end, a deployment monitoring plan for ASAQ Winthrop was designed to provide quality efficacy and safety data through a series of interventional and observational studies [20]. As part of this programme, the present study was initiated in the Agboville health district in Côte d'Ivoire parallel with a large ASAQ implementation survey [21]. The objective was to monitor the effectiveness of ASAQ Winthrop in the treatment of uncomplicated malaria under real-world conditions of care over a three-year period in order to detect any loss of activity that may indicate the development of resistance.

## 2. Methods

### 2.1. Study Design

This was an open-label Phase IV hypothesis-testing study performed in a single health centre (HC) at Grand Morié in the Agboville district of Côte d'Ivoire. The study was conducted in two parts using an identical design over two distinct enrolment periods separated by an interval of three years, the first from November 2009 to May 2010 and the second from March to October 2013. These two periods corresponded to the beginning and end of a programme of systematic implementation of ACT with ASAQ Winthrop in the health district [21]. The investigator in charge of enrolment and patient follow-up and evaluation was the same during both study periods. Each study period lasted six months. This was considered sufficient time to enrol the needed number of patients and to follow the last included patients for up to 28 days or up to the date of treatment failure if observed earlier. During each study period, patients with uncomplicated* P. falciparum* malaria were enrolled in the study and followed for 28 ± 2 days, with six planned study visits at D0 (inclusion visit and 1st treatment day), D3 (day after end of treatment), D7 ± 1, D14 ± 1, D21 ± 1 (for follow-up), and D28 ± 2 (end of study visit).

The study was conducted in collaboration with the NMCP of Côte d'Ivoire, the* Institut Pasteur de Côte d'Ivoire* for parasitological assessment during the first period, the* Centre de Diagnostic et de Recherche sur le Sida et les Infections Opportunistes* (CeDReS; Abidjan, Côte d'Ivoire) for parasitological assessment during the second period and the* Institut de Recherche Biomédicale des Armées* (IRBA; Marseille, France) for genotyping.

### 2.2. Inclusion Criteria

The study included children (≥5 kg body weight) and adults presenting with proven monospecific infection to* P. falciparum* with parasitaemia >2000/*μ*L on blood smears, axillary temperature ≥37.5°C at inclusion or within the past 24 hours, as recommended in the WHO diagnostic criteria [22]. Participants were required to provide signed informed consent and to be able to take treatment orally. For children, informed consent was provided by a parent or guardian. Women of child-bearing age could only participate if they had a negative pregnancy test prior to treatment initiation. Exclusion criteria included complicated or severe malaria, severe comorbidities, known allergy or intolerance to artesunate or amodiaquine, visual disorders suggestive of retinopathy, comedication with drugs that may interact with artesunate or amodiaquine, and previous treatment with an effective antimalarial drug in the previous fourteen days. The definitions of uncomplicated and severe malaria followed WHO guidelines [23].

### 2.3. Treatment

All patients received ASAQ Winthrop using the recommended treatment regimen. ASAQ Winthrop was provided as oral tablets which were to be taken once a day for three days. Administration of the first dose of ASAQ Winthrop was supervised. Tablets could be dissolved in a small amount of water. The dose was adjusted according to the patient's age using three homothetic dosage strengths (AS 25 mg/AQ 67.5 mg one tablet/day for 2–11 months; AS 50 mg/AQ 135 mg one tablet/day for 1–5 years; AS 100 mg/AQ 270 mg one tablet/day for 6–13 years and two tablets/day for ≥14 years). Compliance was evaluated by counting unused tablets in the packet returned on D3 and by asking the patient.

### 2.4. Data Collection

At the baseline visit, demographic features (age, gender, and weight), past medical history, concomitant medication, and clinical features presented were documented, and a fingerprick blood sample taken for smear tests using a vaccinostyle. At each follow-up visit, a physical examination was performed, clinical signs and symptoms documented, and a fingerprick blood sample taken. Blood samples were taken at Visits D7 and D28 (and at D14 in case of abnormal values on D7) for liver and renal function tests and blood cell count.

### 2.5. Determination of Parasitaemia

Thin and thick blood smears were prepared and stained with a May-Grünwald-Giemsa solution. All slides of blood smears were read by qualified personnel from the Institut Pasteur for Period 1 and from the CeDReS for Period 2 according to standard laboratory procedures. Slides were considered negative if no parasite was detected after reading 200 high-powered fields. The presence and density of gametocytes were also determined.

Blood spots on Whatman 3 M filter paper (four spots per card) were prepared for polymerase chain reaction (PCR) genotyping for all subjects at D0 and at the relevant follow-up visit in the case of treatment failure. Samples were frozen and stored at −20°C before genotyping. Two spots were reserved for molecular genotyping, which was performed centrally at the* Institut de Recherche Biomedicale des Armées* (IRBA), Marseille, France [24].

### 2.6. Efficacy Evaluation

Treatment outcomes were classified according to the WHO criteria as adequate clinical and parasitological response (ACPR), early or late clinical failure and early or late parasitological failure at Day 28 confirmed by PCR [23, 24]. Secondary outcomes included the proportion of patients who were afebrile at Day 3, the proportion of patients who were free from parasites at Day 3, and the evolution of gametocyte carriers and mean gametocyte load over the course of the follow-up.

### 2.7. Safety Evaluation

Safety was evaluated through documentation of adverse events (AEs). These were coded using the Medical Dictionary for Regulatory Activities (MedDRA). The investigator asked patients about the occurrence of any adverse event at each study visit. In addition, standard liver and renal function tests and blood cell counts were performed on blood samples taken at D0, D3 (haemoglobin only), D7, D14 (only if anomalies had been observed on D7), and D28. Additional laboratory tests could be performed if required at the investigator's discretion.

Adverse events of special interest were neutropenia, hepatic dysfunction, and symptoms suggestive of extrapyramidal disorders or retinopathy. Neutropenia was defined as a neutrophil count <400/mm^3^ for children or <750/mm^3^ for adults. Hepatic dysfunction was defined as a level of serum alanine aminotransferase (ALAT) >8 × the upper limit or normal (ULN) or >3 × ULN together with total serum bilirubin >2 × ULN.

### 2.8. Statistical Analysis

This study tested the null hypothesis that the ACPR observed during the second study period would be inferior to that observed during the first period. The target sample size was defined a priori in order to evaluate noninferiority with the desired precision. It was hypothesized that noninferiority could be concluded if the difference in ACPR rates between the two study periods did not exceed 5% (i.e., if the lower bound of the 95% confidence interval was superior to a prespecified noninferiority margin of −0.05). Assuming an ACPR rate of around 96%, as observed in previous studies of ASAQ Winthrop elsewhere in Africa [13, 15, 25], it would be required to evaluate 262 patients in each period in order to demonstrate noninferiority with a one-sided *α*/2 risk of 2.5% and a power of 80%. Taking into account an anticipated premature study discontinuation rate of 10%, 580 patients were planned to be included as a whole (290 patients per period group).

Three study populations were determined. The safety population consisted of all study patients having received at least one treatment dose. The intent-to-treat (ITT) population consisted of all patients in the safety population who did not reject the first administered dose. The per protocol (PP) population consisted of all patients in the ITT population without major protocol deviations who completed the protocol as planned. The primary analysis was performed in the PP population. All other efficacy analyses were performed in the ITT population and the safety analysis was performed in the safety population.

### 2.9. Ethics

The study protocol was submitted to the Ethics Committee* (Comité National d'Éthique et de la Recherche)* of the Republic of Côte d'Ivoire for review and written approval, as well as to an Ethics Committee in France* (Comité de Protection des Personnes d'Ile de France XI)* for review and comments. The study was carried out in accordance with International Conference on Harmonization Guidelines for Good Clinical Practice and the Declaration of Helsinki and with the laws and regulations, as well as any applicable guidelines, of the Republic of Côte d'Ivoire. For children, informed consent was provided by a parent or guardian. For patients who were illiterate, study information was read and translated if appropriate, to the patient in the presence of a witness, who signed the form on behalf of the patient if the latter agreed to participate.

## 3. Results

### 3.1. Patient Disposition

**Figure 1:**
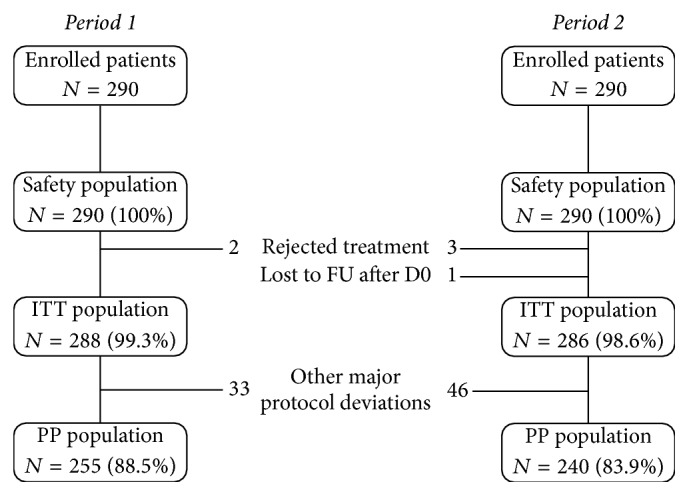
Trial profile. Percentages are calculated with respect to the previous line in the flow chart. FU: follow-up; ITT: intention to treat; PP: per protocol.

Patient disposition across the two periods of the study is illustrated in Figure 1. Overall, 290 patients were enrolled during each study period. All study patients received at least one treatment dose (including patients having rejected the first administered dose twice). These constitute the safety population. Two patients during Period 1 and three during Period 2 rejected or vomited the study treatment and one patient during Period 2 was lost to follow-up after the inclusion visit. The remaining 288 (Period 1) and 286 (Period 2) patients constituted the ITT population. Eighty-five patients (including the six patients excluded from the ITT population) presented at least one major protocol deviation and were thus excluded from the PP population. These protocol deviations consisted principally of treatment administration not in accordance with the protocol (26 patients: 7 during Period 1 and 19 during Period 2) or lack of efficacy assessment at D28 (31 patients: 9 during Period 1 and 21 during Period 2). The total number of patients with major protocol deviations was somewhat higher during Period 2 (50 patients; 17.2% of the enrolled population) than during Period 1 (35 patients; 12.1%). The PP population consisted of 255 patients in Period 1 and 240 patients in Period 2.

### 3.2. Patient Characteristics

Patient characteristics at inclusion are presented in Table 1. The demographic features of the study population and the presence of clinical signs and symptoms were similar between Period 1 and Period 2. Mean parasite density was significantly higher during Period 2 than during Period 1 (*p* < 0.001; Student's* t*-test). A small minority of patients (<5%) were gametocyte carriers at the inclusion visit.

### 3.3. Treatment Compliance

Administration of the first treatment dose was supervised. Nonetheless, five patients rejected or vomited their medication at the first intake and a further 44 patients (6.3%; 18 during Period 1 and 26 during Period 2) were not compliant, failing to take all of the planned daily doses.

### 3.4. Effectiveness

**Figure 2:**
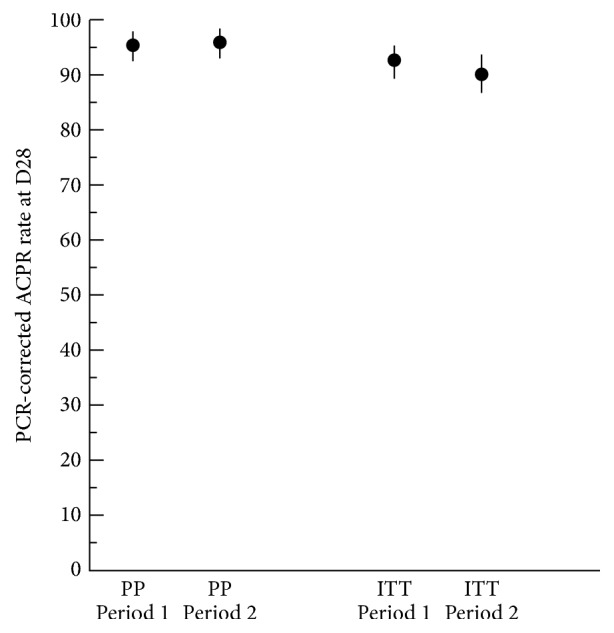
Adequate clinical and parasitological response (ACPR) rates at Day 28 after PCR correction in the per protocol (PP) and intention-to-treat (ITT) populations. ACPR rates are presented with their 95% confidence limits.

The primary effectiveness variable was the ACPR rate at D28 in the PP population after PCR correction (Table 2; Figure 2). Overall 475/495 treated patients in the PP population achieved an ACPR (95.7% during Period 1 and 96.3% during Period 2). Effectiveness could not be assessed in eight patients enrolled during Period 1 and in six patients during Period 2 since a valid PCR analysis of* Plasmodium* DNA could not be obtained from the dried blood spot. The difference in ACPR rates between the two periods was 0.6%. Since the lower limit of the 95% CI [−0.029; 0.040] of the difference in ACPR rates was superior to the prespecified noninferiority margin of −5%, it was concluded that the efficacy of ASAQ during Period 2 was noninferior to its efficacy during Period 1. The null hypothesis was thus rejected. Three patients were identified with late clinical failure during Period 1 and two patients during Period 2; in addition, one patient was identified with late parasitological failure during Period 2.

The overall ACPR rate (both periods combined) was 96.0 [95% CI: 93.7–97.5%]. The ACPR rate was similar in children under five (95.0% in Period 1 and 96.1% in Period 2) and in older children and adults (97.3% in Period 1 and 96.7% in Period 2).

Secondary outcome variables in the ITT populations from the two study periods are presented in Table 3. These were essentially similar between the two study periods. Noninferiority of ACPR after PCR correction during Period 2 compared to Period 1 could not be demonstrated formally in the ITT population; this may be explained by the higher number of nonassessable patients without efficacy assessment in Period 2 than in Period 1 (18 versus 7), which meant that less information on efficacy assessment was available in Period 2 than in Period 1. At D3, thirteen patients during Period 1 and four during Period 2 presented residual fever and only one patient during Period 2 (and none during Period 1) had detectable parasites in thick blood smears. At D28, all patients had successfully eliminated gametocytes during both treatment periods.

### 3.5. Safety

Overall, 259 (44.7%) patients experienced at least one AE during the study (Table 4). However, only nineteen AEs in nineteen patients (3.3%) were considered as possibly related to the study treatment by the investigator. These AEs were vomiting in six patients, abnormal liver function tests and pruritus in three patients each, hypersomnia in two patients, and salivary hypersecretion, somnolence, nystagmus, vertigo, and decrease of appetite in one patient each. No signs or symptoms suggestive of an extrapyramidal disorder or of retinopathy were reported. There was no obvious difference in the nature or frequency of adverse events between the two treatment periods. Of the 325 reported AEs, 84 were mild in intensity, 223 were moderate, and 18 were severe. Seven of the severe AEs were considered potentially treatment-related, namely, three cases of vomiting, two cases of pruritus, and one case each of abnormal liver function tests and decreased appetite.

Six serious AEs were reported in five patients; these were abnormal liver function tests in three patients, which was associated with dysentery in one patient, and anaemia and febrile convulsions, reported in one patient each. Only the cases of abnormal liver function tests were considered as possibly related to the study treatment by the investigator. No deaths and no adverse events leading to treatment discontinuation were reported.

Regarding laboratory safety, no cases of neutropenia (as defined above) were identified. During the first period, three patients were identified with ALAT values >3 × ULN associated with a total serum bilirubin >2 × ULN which were considered as potentially related to treatment. Two of these cases recovered and one was recovering at the end of the study.

### 3.6. Discussion

The principal objective of the study was to compare the effectiveness of ASAQ Winthrop, as measured by the ACPR, at the beginning and end of a three-year period, over which an extensive programme of ASAQ Winthrop was implemented in the area [21]. This is an important aim in order to identify any reduction in effectiveness that may indicate emerging resistance, as a result of possible high drug pressure during the implementation period. Between the beginning of the first study period and the end of the second, ASAQ Winthrop was the first-line antimalarial treatment in Côte d'Ivoire and specific efforts were made to ensure its constant availability to public HCs in the district. No reduction in the efficacy of ASAQ Winthrop was observed between the two study periods. Similar levels of parasite elimination were achieved in spite of the fact that parasite load was higher during Period 2 than during Period 1. This difference in parasitic load may be due to the fact that Period 2 corresponded to the rainy season, whereas Period 1 mainly corresponded to the dry season. The findings of this study can be compared with a similar effectiveness study reported from Nigeria [26], in which no evidence for the emergence of resistance was found in children in the five years following the introduction of ACT (ASAQ or artemether-lumefantrine) as the reference malaria treatment in this country. Similarly, in Tanzania, episodic effectiveness studies have failed to find clinical evidence for resistance to artemether-lumefantrine since this ACT was introduced as a standard first-line treatment of uncomplicated malaria in 2006 [27].

Moreover, the overall ACPR rate (both periods combined) was 96%, which is consistent with those reported from randomised clinical trials [13, 15, 25]. This suggests that high levels of effectiveness can be achieved in the real-world treatment setting.

With regard to safety, no unanticipated adverse events were observed and the incidence of serious adverse events was ≤1%. Three of the six reported serious adverse events related to abnormalities in liver function tests, which are characterised adverse events associated with amodiaquine. No cases of neutropenia, the other principal adverse events of interest, were observed. The incidence and nature of adverse events in this study was comparable to that reported in other randomised clinical trials of ASAQ [13, 15, 25].

The number of major protocol deviations (85 patients, 14.7% of all enrolments) was relatively high. These corresponded principally to inappropriate treatment administration or to lack of efficacy assessment at D28. The former type of violation probably reflects the fact that treatment administration after the first dose was unsupervised. The second type of violation was higher during the second period than the first and may be related to the frequent public holidays that occurred during the second period, leading to patients travelling away from home to visit friends or relatives.

This study has a number of strengths and limitations. The principal strength relates to the fact that an identical design was used for the two periods of the study, which were performed at the same centre HC by the same investigators. This should ensure comparability of the findings over the three-year period that separated the two study periods. The principal limitation relates to the absence of supervised drug administration; only the first treatment intake was supervised, which means that certain patients may not have respected the recommended dosage regimen, leading to suboptimal elimination of the parasite. Nonetheless, despite unsupervised administration on the second and third treatment days, estimated compliance with treatment was good with 93.8% of patients during the first study period and 90.9% during the second period being fully compliant. Finally, the observation period of the study (28 days) was shorter than that recommended in current WHO guidelines [28] which propose a 43-day follow-up period for this kind of effectiveness surveillance study. However, this guideline had not been issued when the protocol of our study was established, and the study was performed using the previous recommended observation period of 28 days. Nonetheless, a more recent study in Côte d'Ivoire using the extended 42-day protocol did not demonstrate any relevant risk of ultra-late (28–42 days) treatment failure with ASAQ and the observed ACPR rate at 42 days was 97% [29].

## 4. Conclusion

High levels of effectiveness and acceptable tolerability were achieved with ASAQ Winthrop fixed-dose combination used for the non-supervised treatment of uncomplicated* P. falciparum* malaria in children and adult patients in Côte d'Ivoire. There was no evidence for loss of effectiveness or for the emergence of resistance to ASAQ Winthrop over the three-year period.

## Figures and Tables

**Table 1:** Patient characteristics at inclusion.

	Period 1	Period 2	*p* value
	*N* = 288	*N* = 286	
Age (years)			
Mean ± SD	5.35 ± 7.63	4.17 ± 4.03	0.483
Median [range]	3 [0.4–62.2]	3.1 [0.3–36.7]	
Children under five years of age	201 (69.8%)	217 (75.9%)	0.101

Gender			0.244
Female	131 (45.5%)	144 (50.3%)	
Male	157 (54.5%)	142 (49.7%)	

Weight (kg)			0.639
Mean ± SD	16.2 ± 11.7	14.8 ± 8.6	
Median [range]	12 [6–71]	12 [5–63]	

Clinical signs and symptoms			
Fever^1^	288 (100%)	286 (100%)	0.163
Asthenia/weakness	241 (83.7%)	234 (81.8%)	0.555
Chills	63 (21.9%)	122 (42.7%)	<0.001
Perspiration	230 (79.9%)	225 (78.7%)	0.725
Headache^2^	61/77 (79.2%)	39/48 (81.3%)	0.783
Pain	18 (6.3%)	25 (8.7%)	0.257
Dizziness^2^	5/73 (6.8%)	7/48 (14.6%)	0.164
Nausea^2^	17/77 (22.1%)	12/48 (25.0%)	0.707
Decreased appetite/anorexia	241 (83.7%)	233 (81.5%)	0.485

Parasitaemia			
Positive thick blood smears^1^	288 (100%)	286 (100%)	NA
Mean parasite density (/*μ*l)	41.6 ± 45.5 (×10^3^)	71.1 ± 85.5 (×10^3^)	<0.001
Gametocyte carriers	7 (2.4%)	5 (1.7%)	0.568
Mean gametocyte density (/*μ*l)	47.1 ± 70.9	967.8 ± 1290.9	0.051

^1^The presence of fever and a positive thick blood smear were obligate inclusion criteria. ^2^Certain symptoms documented from self-report, such as nausea, headache, and dizziness could not be ascertained in young infants.

**Table 2:** Effectiveness: treatment response in the per protocol population.

	Period 1	Period 2
	*N* = 255	*N* = 240
Adequate clinical and parasitological response (ACPR)	244 (95.7%)	231 (96.3%)
Early clinical failure	None	None
Early parasitological failure	None	None
Late clinical failure	3 (1.2%)	2 (0.8%)
Late parasitological failure	None	1 (0.4%)
Not assessable	8 (3.1%)	6 (2.6%)

ACPR and parasitological failure were confirmed by PCR. The proportion of patients with ACPR was not significantly different between the two treatment periods (*p* = 0.82; Fisher's exact test).

**Table 3:** Secondary effectiveness outcome variables in the ITT population.

	Period 1	Period 2	*p* value
	*N* = 288	*N* = 286	
ACPR rate at D28 after PCR correction	267 (92.7%)	259 (90.6%)	0.385
Absence of fever at D3	271 (95.4%)	274 (98.6%)	0.046
Parasite clearance at D3	284/284^1^ (100.0%)	276/278^1^ (99.3%)	0.484
Mean time to parasite clearance (days)	3.0 ± 0.1	3.0 ± 0.2	0.031
Gametocyte carriers at D28	None	None	—

^1^Information on parasitaemia was missing for 4 patients in Period 1 and for 8 patients in Period 2.

**Table 4:** Overview of adverse events.

	Period 1	Period 2	*p* value
	*N* = 290	*N* = 290	
Any adverse event	145 (50.0%)	114 (39.3%)	0.010
Potentially ASAQ-related adverse events	14 (4.8%)	5 (1.7%)	<0.001
Serious adverse event	3 (1.0%)	2 (0.7%)	1.000
Severe adverse events	8 (2.8%)	8 (2.8%)	1.000
Deaths	None	None	—
Adverse events leading to treatment discontinuation	None	None	—

Data are presented as the number of patients presenting at least one adverse event.
